# Supplementary material for: Effect of Relative Humidity on Transfer of Aerosol-Deposited Artificial and Human Saliva from Surfaces to Artificial Finger-Pads
Source: Viruses. 2022 May 15;14(5):1048. doi: 10.3390/v14051048 (PMC9146372; doi:10.3390/v14051048)
Supplement: Supplementary file 1 [file viruses-14-01048-s001.zip › viruses-1712088-supplementary.pdf]

Supplementary information

Effect of relative humidity on transfer of aerosol deposited artificial and human saliva from surface to artificial finger-pad

1. Physical properties of coupons

Coupons were purchased from and cut by Stead & Wilkins (https://www.steadandwilkins.co.uk/) to dimensions indicated in Table S1 with 2% tolerance (50 mm length, 25 mm wide, and 1.5 mm depth; 12.5 cm^2 surface area). There was slight variation in surface area across coupon types with a mean of 12.92 ± 0.031 cm^2 (CV = 2.24%). Mechanical properties are indicated below including, ultimate tensile strength (Rm) and yield strength (Rp).

<u>Stainless steel 304</u>: Composition (C – 0.023%, Cr – 18.0%, Mn – 1.85%, Ni – 8.0%, P – 0.035%, S – 0.01%, Si – 0.35%). Physical properties (Rm – 610 N/mm^2, Rp 0.2 – 301.8 N/mm^2, Rp 1.0 – 335.4 N/mm^2, A80 – 59.5%). Note: the fine shot blasted surface was used for contact experiments.

<u>Aluminium alloy 5251</u>: Temper H22 (strain hardened, partially annealed, ¼ strength). Composition (Si – 0.14%, Fe – 0.35%, Cu – 0.05%, Mn – 0.39%, Mg – 1.9%, Cr – 0.02%, Zn – 0.02%, Ti – 0.01%). Physical properties (Rm – 214 MPa, Rp 0.2 – 178 MPa, A50 – 10%).

<u>KYDEX®-T thermoplastic</u>: https://kydex.com/products/kydex-t/ High-impact fire rated sheet of PVC/PMMA alloy. Specific gravity – 1.35 g/cm^3; tensile strength – 42 MPa (6100 psi); flexural strength – 66 MPa (9600 psi); modulus of elasticity – 2400 MPa (360,000 psi); Rockwell hardness, R scale – 94; Heat deflection temperature – 75.6 °C. Note: the Haircell textured embossed shiny side was used for contact experiments.

<u>Melamine laminate</u> (high pressure): ABET rigid engraving laminate; https://www.engraving-supplies.co.uk/rotary-materials/plastic/abet-rigid.html. Layers of kraft paper impregnated with phenolic resins with outer layers of decorative paper impregnated with aminoplastic resins, bonded together by high pressure (9 MPa) and temperature (150 °C). Specific gravity – 1.35 g/cm^3; tensile strength – 100 MPa; flexural strength – 100 MPa; modulus of elasticity – 10,000 MPa.

<u>ABS (smooth)</u>: https://epsotech.com/en/; Epsotech AB AN3 NC is a single or multi-layer product made from ABS with a semi-matt surface aspect. Specific gravity – 1.1 g/cm^3; tensile strength – 42 MPa; modulus of elasticity – 2100 MPa; elongation at yield – 3%; VISCAT softening point – 107 °C; heat deflection temperature – 90.0 °C; thermo-forming temperature – 180 – 220 °C. Note – the non-semi-matt side (i.e shiny side) was used for contact experiments.

<u>ABS (pinseal)</u>: https://epsotech.com/en/; Super high impact B400 series with embossed surface. Specific gravity – 1.06 g/cm^3; tensile strength – 36MPa; flexural strength – 50 MPa; VISCAT softening point – 97.0 °C; heat deflection temperature – 97.0 °C. Note: the Pinseal textured embossed shiny side was used for contact experiments.

Table S1. Measured coupon dimensions.

Coupon material†	No. coupons	Dimension	Statistical parameters					
			Min	Max	Mean	S.E Mean	Variance	CV
SS304	16	Width (mm)	24.90	25.91	25.16	0.061	0.006	0.97
		Length (mm)	49.90	49.98	49.94	0.007	0.0009	0.06
		Area (cm^2)	21.44	21.95	12.57	0.031	0.015	0.98
ABSS	16	Width (mm)	25.81	26.16	25.99	0.030	0.015	0.47
		Length (mm)	50.07	50.50	50.33	0.025	0.0098	0.20
		Area (cm^2)	12.96	13.18	13.08	0.018	0.0051	0.55
ABST	16	Width (mm)	24.80	26.36	25.64	0.101	0.163	1.58
		Length (mm)	49.73	50.45	50.28	0.052	0.042	0.41
		Area (cm^2)	12.50	13.27	12.89	0.048	0.036	1.48
ALU	16	Width (mm)	25.16	25.37	25.26	0.014	0.0033	0.23
		Length (mm)	50.03	50.20	50.10	0.011	0.0019	0.09
		Area (cm^2)	12.59	12.72	12.66	0.009	0.0013	0.28
KYD	12	Width (mm)	25.83	26.17	26.04	0.032	0.0122	0.42
		Length (mm)	50.16	50.54	50.43	0.031	0.0115	0.21
		Area (cm^2)	12.97	13.20	13.13	0.021	0.0053	0.55
MEL	12	Width (mm)	26.16	26.77	26.46	0.059	0.041	0.77
		Length (mm)	50.29	50.54	50.44	0.020	0.0049	0.14
		Area (cm^2)	13.22	13.48	13.35	0.025	0.0075	0.65
Total	88	Width (mm)	24.80	26.77	25.71	0.053	0.249	1.94
		Length (mm)	49.73	50.54	50.24	0.022	0.044	0.42
		Area (cm^2)	12.44	13.48	12.92	0.031	0.084	2.24

†SS = stainless steel 304 (0.9 mm depth), ABSS = ABS plastic (smooth; 1.5 mm depth), ABST = ABS plastic (textured; 1.5 mm depth), ALU = aluminium alloy 5251 (1.0 mm depth), KYD = KYDEX (plastic; 1.5 mm depth), MEL = melamine laminate (plastic; 1.7 mm depth)

2. Production of artificial finger-pads

A molding process was developed for generation of replica finger-pads with faithful representation of topological features such as ridges and furrows of finger surface. The index finger of an adult male subject's right hand was used. Briefly, initially a master cast and mold was generated. Degassed Dragon Skin™ FX-Pro silicone (DDS; 2A Shore hardness; Bentley Advanced Materials, UK) was applied as a slip coat to a volunteers left middle finger pad. Once cured the slip-coated finger is placed onto a foundation slab of DDS within a 3D-printed tray designed to support, position and flatten the finger to a constant contact area. DDS is then poured to a set level providing a top coat and allowed to solidify. Removal of the finger leaves a negative master mold into which resin (Smooth-Cast™ 310, Smooth-On, UK) is poured and after solidification produces a negative master cast, machined and built up to provide substrate for an integrated flange. DDS was degassed within a vacuum degassing system (DS26-P, EasyComposites, UK).

For batch production, six negative polyurethane casts are prepared and fixed into position in a 3D-printed tray. DDS is poured into the tray to a determined level and allowed to solidify generating a six finger-pad negative mold in DDS. The negative mold is clamped into a new 3D-printed tray with a slope. Magnetic perforated steel mesh (2 mm hole, 0.7 mm depth; RS Pro, UK) cut to dimension of the finger-pad base is added to provide both support to the finger compression (i.e. represents distal phalanx) and means of magnetic attachment to the force-rig mount. Individual identifier numbers are also added. DDS is poured into the tray using the slope and allowed to solidify providing six identical finger-pads with embedded metal backing and unique identifier.

Individual finger-pads have dimensions of 41 × 22 mm (13 mm depth). After production of a batch the finger-pads are translucent and oily to touch due to presence of silicone oils developed during curing. Excess silicone oils are removed from the finger-pads by vigorously wiping the surface with two cyclohexane (Sigma-Aldrich, UK) soaked wipes (TORX Xpress 10 02 88, Essity UK Ltd) then wiping dry, before ageing over a couple of weeks with further removal of excess oils until the finger-pads become slightly opaque. A non-stabilized artificial eccrine sweat-sebum emulsion (Verulam Scientific Ltd, UK) was applied to each finger-pad before use in contact transfer experiments. Four coats were required using multiple passes of a emulsion soaked foam bud (RS Pro, UK) with drying between each application to ensure complete coverage and provide surface wetting properties consistent with a human finger. Each application involved multiple passes over each AFP varying the sequence of AFP's and reloading of the bud.

Figure S1. Schematic of experimental aerosol deposition and touch transfer process

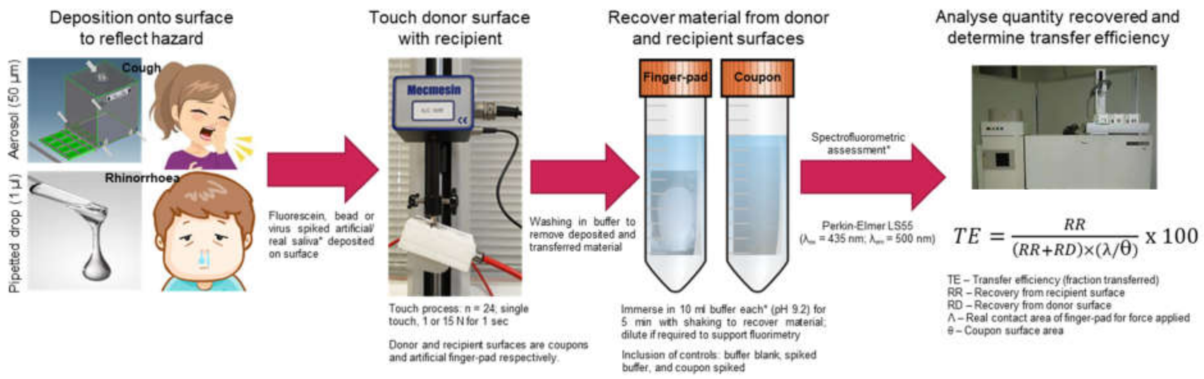

3. Understanding impact of flow and spatial patterns within deposition apparatus

The aerosol deposition apparatus is described in detail in Section 2.2 of Materials and Methods. Figure S1 shows the key features of the apparatus. Initial assessments of deposition consistency were conducted by spraying a 1 g·L^{-1} fluorescein solution (50:50 ethanol-water mixture) onto stainless steel coupons at 50% RH. Coupons were positioned at identical places within the trays for each experimental spray (Fig. S2a). Impaction of droplets on the top of the box was observed indicating that the fan mounting required positioning lower within the apparatus (Figure S2b). Table S2 demonstrates the iterative approach to understanding spatial variation within the deposition box and incremental improvements to deposition on coupons placed within the apparatus permitting coupon-to-coupon variation to be reduced from 19.4 to 8.6% CV (Figure S3). Rows A to I represent assessments of aerosol mixing on variability in spatial deposition. Rows A to G represent movement and alignment of mixing fan (3 V) to optimise positioning, reduce impaction on box walls and improve spatial distribution. Row H and I demonstrate consistent aerosol deposition with acceptable variation (~10% CV) due to optimal positioning and an improved mixing fan (i.e. change from 3 to 6V fan).

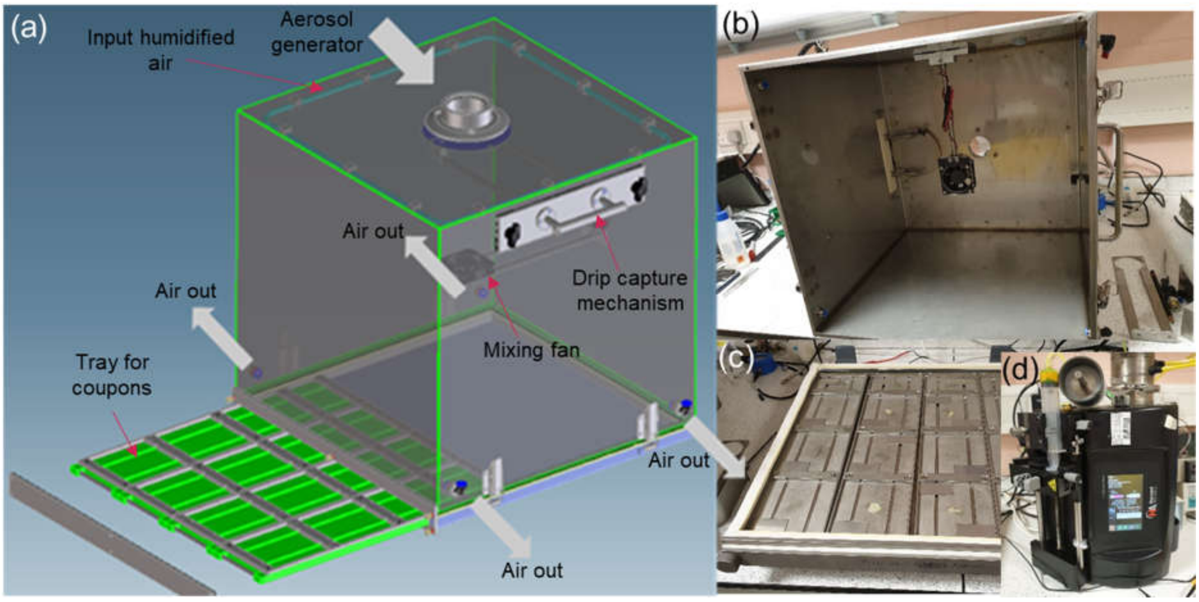

Figure S2. Experimental apparatus for surface deposition of large aerosol droplets and particles. (a) Design schematic, (b) internal of deposition box, (c) tray system with steel coupons positioned, and (d) Sono-tek ultrasonic nozzle and syringe drive.

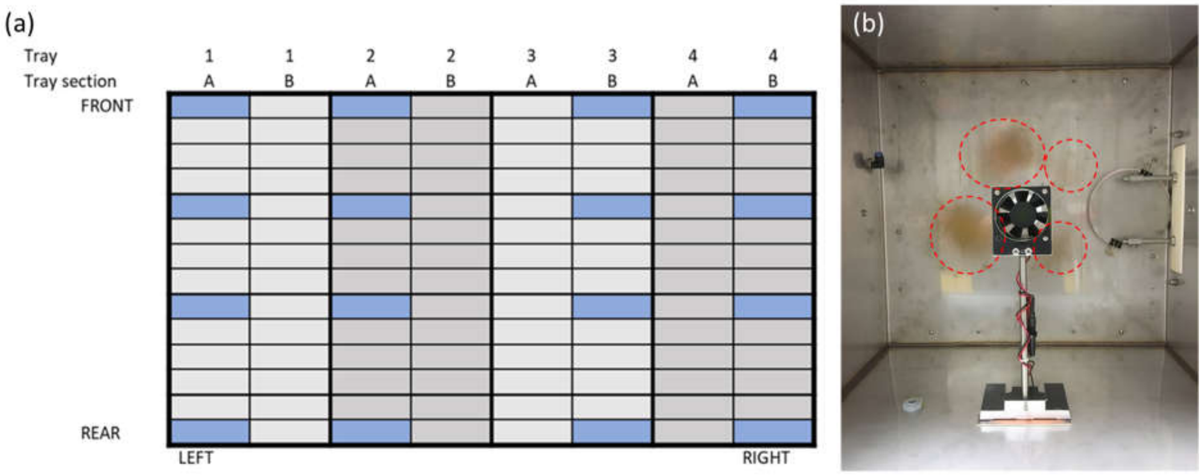

Figure S3. Characterisation of deposition apparatus. (a) Spatial positioning of stainless steel coupons across the four trays. Blue shaded boxes indicate position of coupons for experimental runs in Table S1, (b) Impaction of droplets on top of deposition box due to fan positioning close to spray nozzle.

Table S2. Spatial variation of aerosol deposition within apparatus.

Experimental run	Fluorescein fluorescence recovered from coupons†					
	Average	S.D	CV (%)	Min	Max	Comment
**A**	293.1	56.7	19.4	219	377	↑ concentration at box rear
**B**	282.1	33.7	11.9	235	356	↑ concentration at box front
**C**	277.9	51.3	18.5	208	359	↑ concentration at box rear
**D**	303.1	51.5	17.0	236	374	↑ concentration at box rear
**E**	297.8	37.9	12.7	242	320	↑ concentration at box rear
**F**	283.9	43.0	15.1	201	358	↑ concentration at box left side
**G**	270.1	38.2	14.1	191	328	Little spatial variation
**H**	243.8	28.6	11.7	204	300	Little spatial variation
**I**	275.8	23.8	8.6	238	325	Little spatial variation

† = fluorescence values should be multiplied by 80 to account for dilution factor from original. Grey rows represent improved system and approach used in transfer experiments.

4. Assessment of artificial saliva aerosol deposition across metal and plastic coupons

Once the system was optimised with respect to air flows, the deposition across a range of coupons types was assessed (Figure S4). Impact of spray fluid was compared by changing to artificial saliva containing 1 $g{\cdot}L^{-1}$ fluorescein for a set of sprays (Table S3). Artificial saliva (with fluorescein) produced greater deposition compared to fluorescein in ethanol alone, however variability was much greater (CV, 27.9%). Significantly greater fluorescence was observed on the plastics, in particular MEL and KYD (54264 ± 9176 and 46464 ± 8400 FU respectively). Assessment with only metals reduced the CV to 6.93% (mean: 53176 ± 3686 FU; n = 52 SS304 and 48 ALU coupons; data not shown). Electrostatic charge effects between plastics and the artificial saliva aerosol particles was considered to be causing the variability. Dissipation of charge on the coupons resulted in reduced variation across all coupon types with total variation of 8.4% CV (mean: 51008 ± 4296 FU). Further to this during later experimental runs the nebulisation was split into four 2.5 minute stages with the coupon trays moved in position and reversed between sprays, this minimised any tray to tray, front to back or side to side variability.

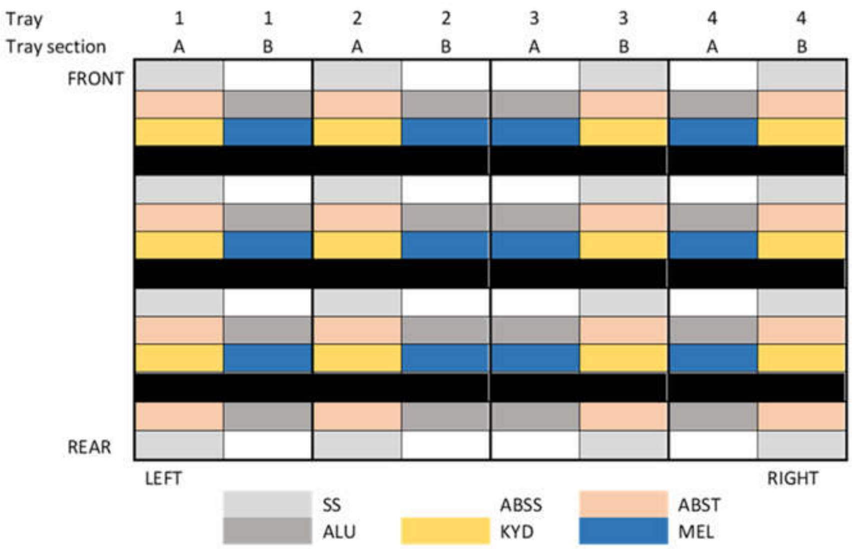

Figure S4. Spatial positioning of coupons across the four trays in deposition box. Blacked shaded boxes represent blanks. Sixteen of each coupon type were assessed, except for KYD (n = 12).

Table S3. Spatial variation of aerosol deposition across different coupon materials as a function of spray fluid composition.

Coupon material	Fluorescein fluorescence recovered from coupons†				
	Average	S.D	Min	Max	CV (%)
**1 g L-1 fluorescein (50:50 ethanol-water mixture)**					
**SS**	316.6	31.1	270.9	382.8	9.82
**ABSS**	380.0	29.4	330.0	421.4	7.74
**ABST**	372.0	49.3	308.9	467.8	13.2
**ALU**	345.9	34.6	294.6	429.0	10.0
**KYD**	365.9	45.4	300.6	439.6	12.4
**MEL**	369.1	41.6	313.8	447.5	11.3
**All coupons**	**357.4**	**43.7**	**270.9**	**467.8**	**12.2**
**1 g L-1 fluorescein in artificial saliva**					
**SS**	297.3	44.0	238.4	404.7	14.8
**ABSS**	475.3	64.7	377.8	605.7	13.6
**ABST**	507.4	84.0	400.5	707.3	16.6
**ALU**	498.3	74.2	388.5	655.0	14.9
**KYD**	580.8	105.0	454.8	759.2	18.1
**MEL**	678.3	114.7	546.4	888.5	16.9
**All coupons**	**494.9**	**138.5**	**238.4**	**888.5**	**27.9**
**1 g L-1 fluorescein in artificial saliva – coupons charge dissipated**					
**SS**	603.3	56.0	490	719	9.3
**ABSS**	663.8	47.8	565	740	7.2
**ABST**	627.8	53.6	484	689	8.5
**ALU**	628.1	39.4	554	686	6.3
**KYD**	640.3	48.6	541	708	7.6
**MEL**	671.8	52.3	574	771	7.8
**All coupons**	**637.6**	**53.7**	**484**	**771**	**8.4**

† = fluorescence values must be multiplied by 80 to account for dilution factor from original.

Particle size distribution as a function of spray fluid is shown in Figure S5. Data taken after 2.5 min of nebulisation starting with the deposition box at 100% RH with mixing fan on. No aerosol background was observed prior to introduction of spray. Sonotek nozzle (25 kHz) was operated according to the Section 2.2 in Methods. Particle measurements were taken with Welas 3000 spectrometer with two 2300 heads (PALAS GmbH). The distributions demonstrate as anticipated the solute composition does not markedly influence the droplet size generated by the nozzle.

Figure S5. Particle size distributions (volume) for artificial saliva, human saliva and water sprayed into deposition box using Welas 3000 spectrometer†. Solid and dashed lines are 100% and 30% RH respectively.

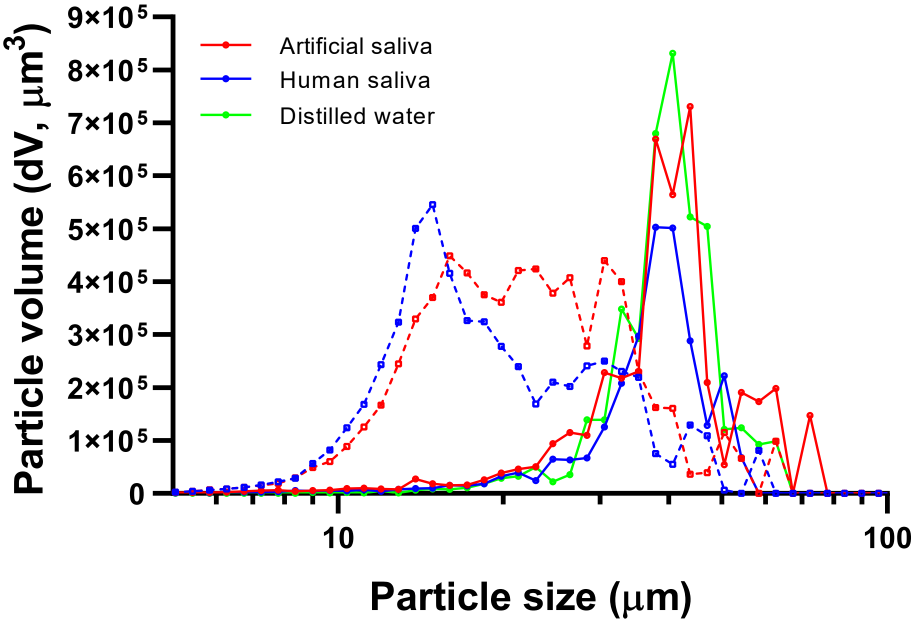

† = Instrument setup was 2000BLDC_MEMS, calibration using Monodust 2000, distribution set to 32 divisions per decade and a measurement range of 2 to 105 μm. Sample flow was set to 5 L·min^{-1}. Refractive index set to 1.33+0i (water). One Welas 2300 head was placed inside the deposition box with a catch pot on its pump tube. Greater than 500 particles were measured for the artificial saliva and human saliva data, and >200 particles for the water spray.

Figure S6 shows deposition patterns and consistency of fluorescein spiked artificial saliva on an example MEL and SS304 coupon. Although the pattern is more difficult to visualise on the SS304 coupon, clear deposits can be observed. The patterning is consistent, and areas of single particle deposition and either multiple impacts onto the same spot or merging of particles that land next to one another can be observed.

After a contact event, particularly as the RH is increased above 40%, features can be seen on the coupon. Figure S7 shows a contact event with an AFP at 65% RH where redistribution of the deposited saliva into distinct masses occurs during the touch event. Interestingly, the material redistributes towards the centre of the area the AFP contacts leaving cleared zone around the edge of the contacted region. Features such as ridge and furrow detail can be observed, and indicates areas where the topology of the AFP prevents redistribution of the saliva.

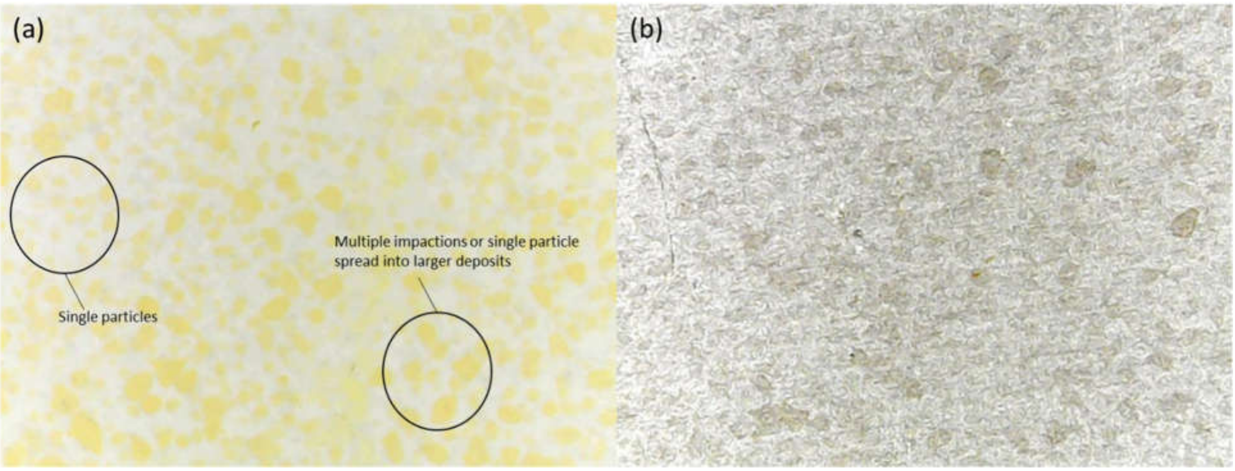

Figure S6. Particle deposition of artificial saliva on (a) MEL and (b) SS304 coupons. Spray was for 10 min into deposition box conditioned at 100% RH. Images take by light microscope at ×10 magnification.

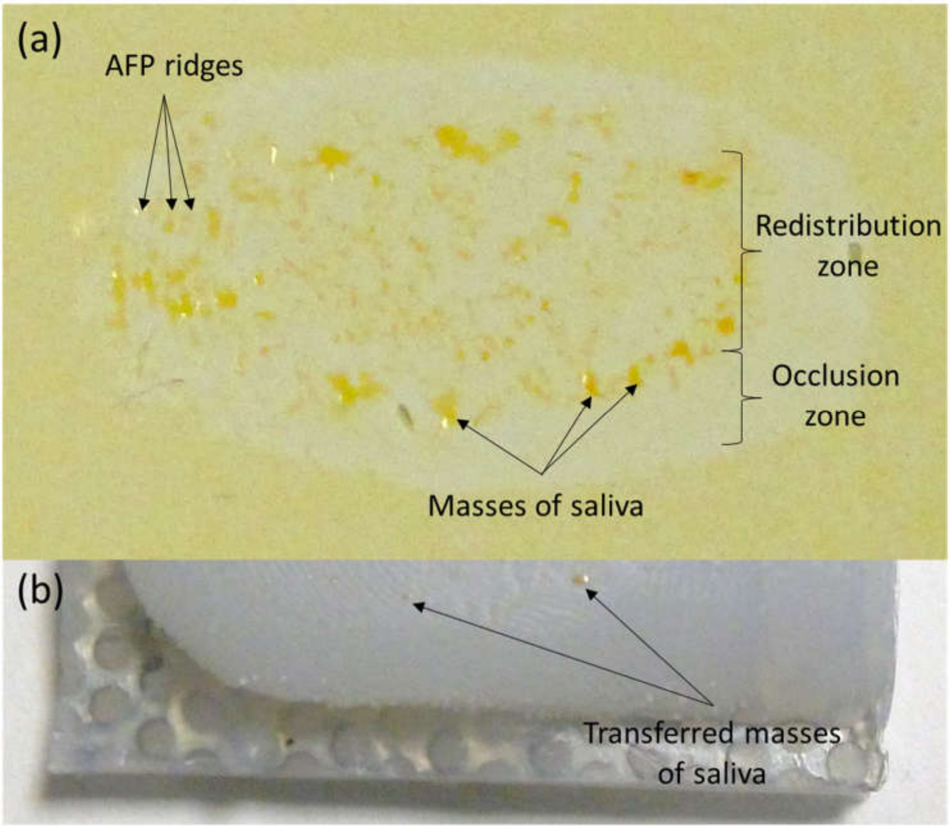

Figure S7. Transfer of human saliva from MEL surface to AFP at 65% RH. (a) MEL coupon showing features within contact area, and (b) AFP showing transferred saliva (image cropped to remove biometric information). Contact event was 15 N for 1 sec. Images take by light microscope at ×10 magnification.

The recovery efficiency of artificial saliva from the range of surfaces used in the study was assessed alongside the AFPs (Figure S8). Irrespective of whether the droplet of artificial salvia was wet or dried

onto the surface recovery was close to 100%. Variability accounted for by dilution and pipetting errors. Similar tests for human saliva did not indicate a difference in recovery between the two deposited materials.

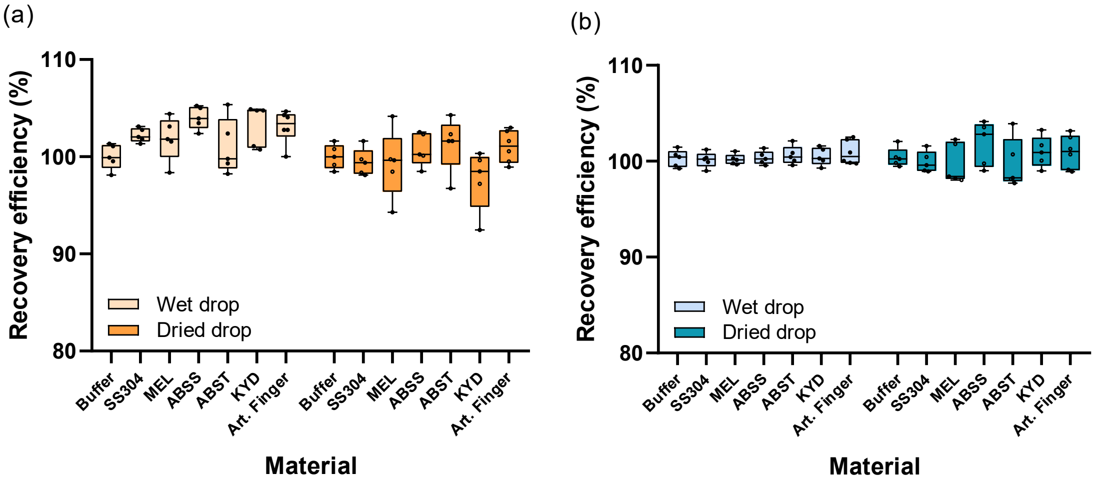

Figure S8. Recovery efficiency of wet and dried droplets (1 µl) from a range of surfaces of (a) artificial saliva and (b) human saliva. Buffer was included as a control, directly spiked with same volume.

5. Contact transfer events

Fig. S9a shows the positioning of the aerosol deposition box and force-rig within the humidified cloche. Wet or dry air flows are pulsed at intervals to reach the required cloche target humidity. Once at the target relative humidity, coupons are removed from the deposition box and permitted to equilibrate to the target humidity for 30 min alongside artificial finger-pads. Fig. S9b shows locality of RH measurements for contact events. The probe is positioned within 50 mm of the coupon surface to display any fluctuations within the cloche due to pulsed mixing of wet or dry air streams with the bulk cloche humidity. The reproducibility of the touch event used in this study is demonstrated in Fig. S10 from a range of experimental runs, where the 15 N touch force is held for a period of 1 second.

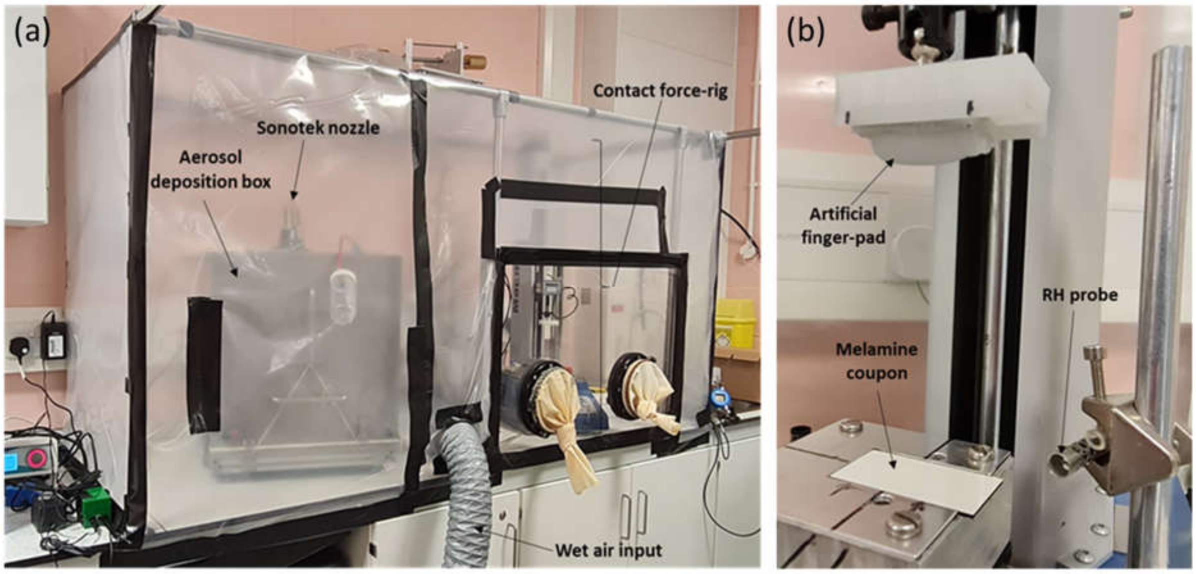

Figure S9. Contact transfer apparatus. (a) Humidified cloche environment, and (b) Close-up of contact event portion of apparatus

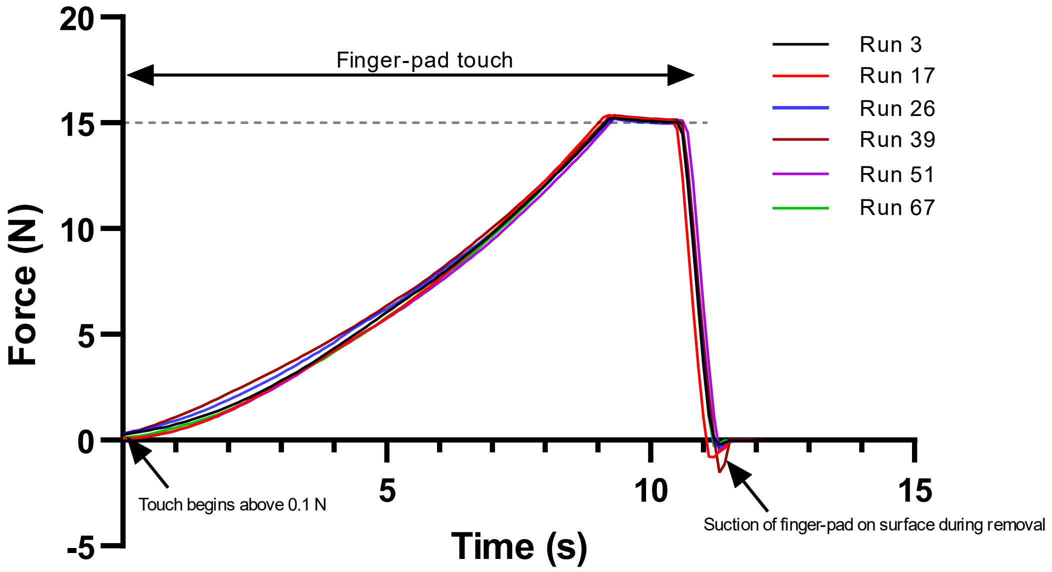

Figure S10. Representative contact events for a 15 N touch for a second.

Figure S11 demonstrates the visible differences in fluid properties of human and artificial saliva. Note that human saliva is a clear solution that readily forms bubbles. Human saliva tended to form a thin layer that gradually tracked back down into the main fluid bulk over time. In contrast, artificial saliva was a visible colloidal suspension and lacked the bubble formation and rheological behaviour displayed by human saliva possibly due to presence of surfactants or other proteins. It may be that the mucins are more readily within solution in human saliva due to biological secretion pathways opposed to the chemical mixing of components during preparation of artificial saliva.

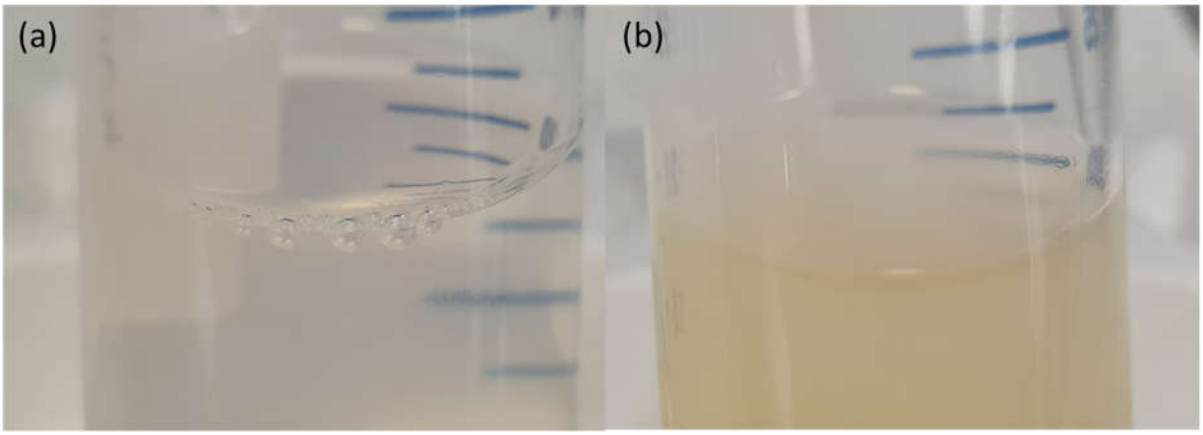

Figure S11. Differences in fluid characteristics of saliva used in this study (a) human saliva, and (b) artificial saliva after 5 seconds shaking.

6. Surface energy measurements

Table S4 shows the measurements obtained across different surfaces for artificial saliva including dispersive and polar components. Table S5 shows dynamic contact angle measurements for the coupons that had different sides. Coupons were fixed together on the sides not evaluated in contact transfer events leaving those which are used in contact events facing outwards and available for assessment in the contact angle measurements. Note the difference between single and double layer coupons for both advancing and receding angle.

Table S4. Surface energy measurements for surfaces and fluids used in the study.

Surface	Sebum	Surface energy ($mN{\cdot}m^{-1}$)		
		Total	Dispersive component	Polar component
**SS304**	-	26.3 ± 0.26	25.0 ± 0.17	1.3 ± 0.09
**ALU**	-	25.8 ± 0.36	25.4 ± 0.28	0.4 ± 0.09
**MEL**	-	29.2 ± 0.52	20.7 ± 0.29	8.5 ± 0.23
**ABSS**	-	28.9 ± 0.23	26.0 ± 0.18	2.9 ± 0.05
**ABST**	-	33.7 ± 0.43	23 .0 ± 0.21	10.7 ± 0.22
**KYD**	-	28.3 ± 0.32	19.4 ± 0.09	9 ± 0.22
**Silicone stub**	-	26.7 ± 0.37	24.4 ± 0.26	2.3 ± 0.11
**Silicone AFP**	-	28.2 ± 0.34	28.2 ± 0.31	0.1 ± 0.03
**Silicone AFP**	+	56.2 ± 0.72	14.0 ± 0.28	42.2 ± 0.44
**Water†**	-	72.8	22.6	50.2
**Hexadecane†**	-	27.5	27.5	0.0
**Ethylene glycol†**	-	48.8	32.8	16.0
**Artificial saliva**	-	64.8 ± 1.5	23.5	41.3
**Human saliva**	-	58.4 ± 1.1	24.5	33.9

† = Probe liquids, water, hexadecane and ethylene glycol are included for comparison.

Table S5. Dynamic contact angle measurements for surfaces when assessed as single and double layered coupons.

Coupon material	Single layer			Double layer		
	Advancing angle	Receding Angle	Difference	Advancing angle	Receding Angle	Difference
**ABST**	90.55	28.28	62.27	73.82	32.14	41.68
**ABSS**	89.05	36.99	52.06	72.19	47.43	24.76
**KYD**	94.1	29.91	64.19	77.46	42.13	35.33

7. Minimum contactable surface area

The contactable area was assessed for SS304 and ABST using a 3D non-contact profilometry technique (focus variation type, Bruker Alicona InfiniteFocusSL, 5x AX objective, IF-MeasureSuite 5.3.6 software). Figure S12 shows surface topography complexity of ABST compared to SS304. Surface scratches can be observed on the SS304 image. Areas were selected in the software across scanned areas where data was most complete. Note that areas of high

reflectance or steep inclines can result in non-scanned spots, visible particularly on the ABST scan as 'white holes' in the image. The projected areas were 6.781 and 9.901 mm2 for SS304 and ABST respectively.

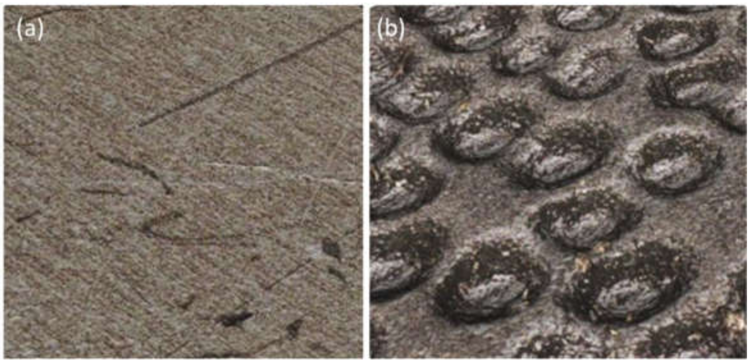

Figure S12. Surface topography scans of coupons (a) SS304 and (b) ABST.

Figure S13 shows slices through the z-plane for the projected area of SS304 and ABST coupons as the plane moved into the surface, from just above the highest point of the surface to positions where increasing surface area is visible. The choice of z-slice thicknesses at which to perform the analysis is a function of the scale of the topography in the z-plane, hence the different measurement steps in Figure S13. This provided a series of 'above surface volume' values that were each divided by the thickness of the corresponding z-slice to provide the 'above surface area' for that particular z-slice. The 'above surface area' was then divided by the projected area for either SS304 or ABST to determine the minimum percentage surface area that could be contacted for a particular z-slice. Figure S14 plots the available surface area as a function of z-plane depth highlighting that SS304 has much more available surface area that can be contacted than ABST, and at a much shallower depth. It also highlights data for a human finger-pad for comparison, showing that the surface area available for contact is similar to ABST. This is explained by the deep furrows and raised ridges of finger-pads.

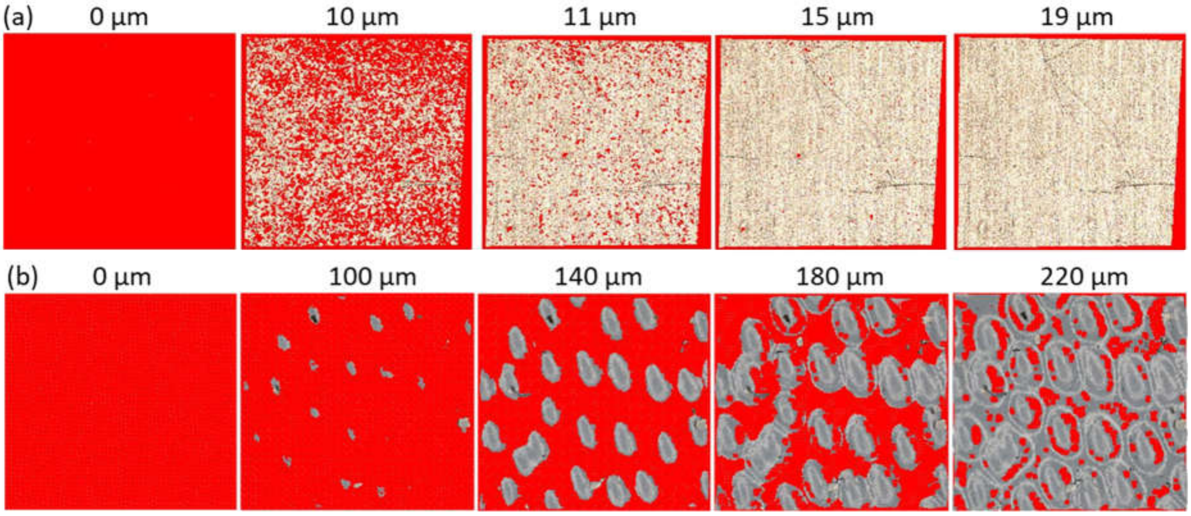

Figure S13. Slices through z-plane for (a) SS304 and (b) ABST coupons. Values represent z-plane depth. Grey indicates area where surface is visible for the particular slice, termed 'above surface volume'.

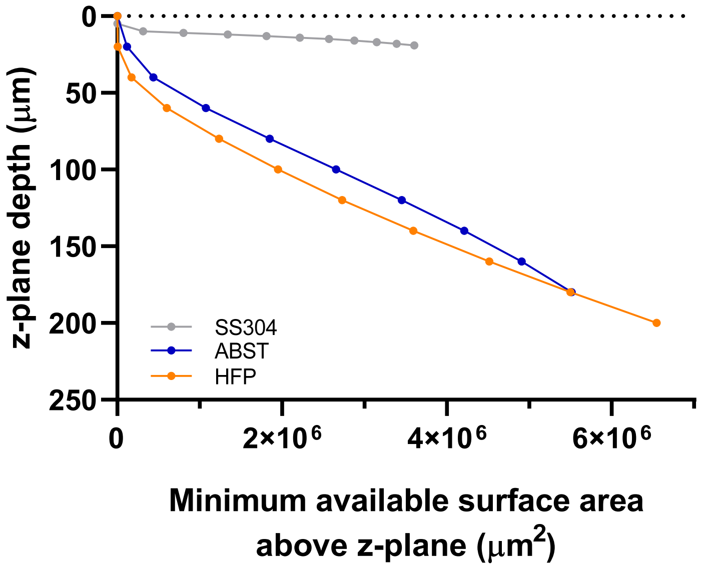

Figure S14. Calculated minimum surface areas above z-plane as a function of depth of z-plane. ABST = ABS pinseal texture, HFP = human finger-pad, SS304 = stainless steel.

8. Relative humidity is a critical factor to control in transfer studies

The large variability in TE's observed in literature studies can be explained by a combination of: (1) the identification in our study of the RH range from 40 to 65% that is associated with rapid changes in drying state of the deposited matrix, and (2) RH dependent survival of the micro-organism on the surfaces [48, 56]. This study evaluated transfer of the deposited matrix thus eliminating any issues with microbial survival.

Stainless steel is an often used material to study transfer efficiency of microorganisms and to a degree can be used to compare between studies [3, 17, 57-63]. Figure S15 compares the TE data for artificial saliva to literature values for a range of viruses and bacteria where stainless steel was used as a contaminated donor surface. Despite differences in how experiments were performed (e.g. touch parameters, volunteers, microbe, suspending matrix, drying state), when accounting for RH ranges used in the various studies, the data for artificial saliva shows similar TE values to many of the studies. Literature studies describing transfers conducted at 40-50% RH all had comparatively low TEs (Figure S15). Only one study looked at a high RH alone, Koenig et al., (2016), with high TE >50% observed for *S. aureus*. The variation observed is likely due the fact RH is difficult to control, for example the study of Ansari *et al*., (1992) reported RH of 50 ± 5%; the range is therefore 45 – 55% and no clarity is presented on the precise RH value for a given contact event. Note the range falls on the steepest part of the curve observed for SS304 in this study (Figure 6a). The TE values of <1% in the study of Ansari et al., (1992) are low which can perhaps be explained by the RH being towards 45% rather than 55%.

Removing consideration of RH within the data in this study increased the variability hugely, with RSDs ranging from 77.5 to 89.4% (Table S6). In contrast, the RSDs for SS304 and MEL when RH is tightly controlled and recorded within the contact process are shown in Table S7 and Fig. S12. At RH 55% and above, the RSDs for both SS304 and MEL are all less than 20%. In contrast at the lower RHs (25 – 50% RH), the RSDs start to increase, ranging predominantly from 20.6 to 50.2%, with an outlier for SS304 at 30% RH (RSD, 84.23%). This is expected as these values fall along the initial part of the curve where small differences in experimentation will have larger effects on the data. However, in relation to the literature where the best studies to date reports RSD of ~35.3% [8], this is an improvement. Indeed Zhao & Li (2021) reviewed the literature and state 'Studies that reported SD values had average RSDs exceeding 40%, and most had values near 100%'. In recent studies on stainless steel using the bacteriophages phi6 and MS2 (RH range 53-71% for steel), transfer efficiencies of 23 ± 19 and 34 ± 12% respectively were observed, providing RSDs of 82.6 and 35.3% [17]. It is likely that some of the variability is due to RH control and monitoring given the dominant effect demonstrated in this study on transfer across a range of surface materials.

A reason for the greater variability at the lower RHs in this study may be local variation in RH within the cloche. Although tightly monitored and recorded at the contact event (Figure S9), the coupons and finger-pads are equilibrated at a different position within the cloche and as the wet or dry air is pulsed in to maintain humidity, there may be local variation that affects the coupon and finger-pads at the microscale level, also any variation in temperature at the cloche walls, floor, etc. could result in localised loss of vapour to those walls before equilibrium is reached. Improvements in mixing of airflows within the cloche and wider monitoring of RH within the cloche, particularly in the area the coupons and finger-pads equilibrate would likely assist in reducing RSDs further. This is less of an issue at RHs above 55% where the transfer efficiency data plateaus for the finger-pad coupon interactions.

The transfer efficiencies and RH ranges examined within the study have applicability to real environments. The usual RH ranges experienced within households across UK and world tend to fall within the range >30 and <70% RH [64-66]. Fig. S16 shows RH sensor data from Pullinger *et al*., (2021) for a single UK house (out of 255). Only the period of August-September was selected due to the large dataset. The mean (and SD) for bathroom, bedroom, hall, kitchen and living room were 65.8 ± 6.6, 62.6 ± 3.8, 62.9 ± 3.9, 60.2 ± 3.4, and 61.2 ± 3.7% respectively. The RH range across all rooms was large (min = 39.3%, max = 99.6%) and spanned the RH values investigated for transfer in this study. Thus, the data generated in this study is representative of both humidity and surfaces found in domestic households.

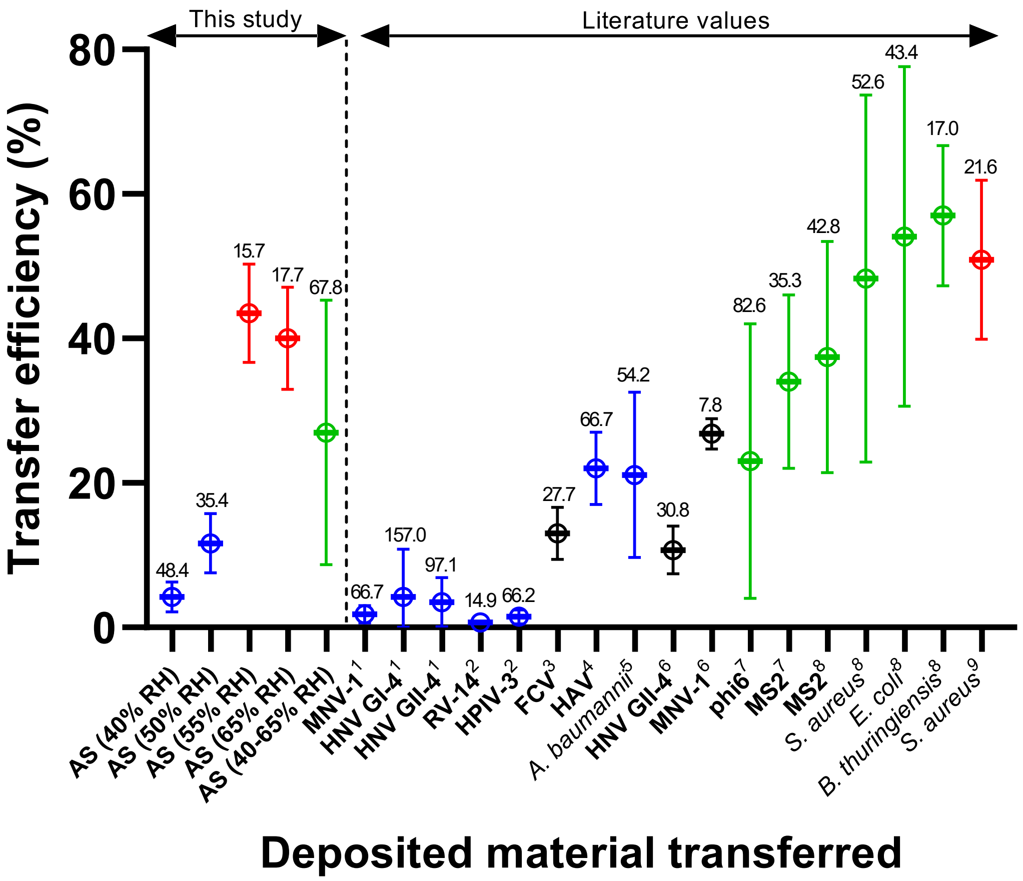

Figure S15. Comparison of transfer efficiencies from stainless steel to finger in this study and literature as a function of relative humidity. Coloured circles represent RH bands as follows: blue = 40-50% RH, red = 55-65%, green = 40 - 65 or 50 - 70% RH, and black = no RH reported. 1 = Tuladhar *et al*., (2013), 2 = Ansari *et al.,* (1991), 3 = Bidawid *et al.,* (2004), 4 = Mbithi *et al*., (1992), 5 = Greene *et al*., (2015), 6 = Stals *et al*., (2013), 7 = Anderson & Boehm, (2021), 8 = Lopez *et al.*, (2013), and 9 = Koenig *et al.,* (2016). Relative standard deviations are denoted above upper error bar.

Table S6. Statistical analysis of transfer efficiency data from this study (artificial saliva across a range of surfaces) and Anderson & Boehm (2020) not accounting for RH. Note values included covered the range 40 – 65% RH only representing the slope in Figure 6a. †= SS304 surface from Anderson & Boehm (2021).

	This study			Anderson & Boehm (2021)	
	SS304	MEL	ABSS	Phi6†	MS2†
**Number of values**	77	83	29	32	30
**Minimum**	1.47	4.3	5.79	2.95	12.59
**Maximum**	56.48	79.95	68.92	87.09	60.74
**Mean**	26.97	41.79	39.71	23.11	34.38
**Std. Deviation**	18.29	22.96	20.77	19.31	12.43
**Relative Std. Deviation**	67.8%	55.0%	52.3%	82.6%	36.2%

Table S7. Statistical analysis of transfer efficiency data from artificial saliva for SS304 and MEL surfaces accounting for RH. All RH values are shown. Tolerances were within 2% of stated RH value. N/A, not applicable

**Coupon material**	**Relative humidity (RH)**								
	25%	30%	40%	50%	55%	65%	75%	85%	Drop
**Stainless steel 304**									
RH (%)	25.22 ± 0.16	30.07 ± 0.21	40.00 ± 0.20	49.95 ± 0.15	55.20 ± 0.11	65.28 ± 0.16	74.98 ± 0.19	85.22 ± 0.44	N/A
Number of values	17	18	18	16	27	16	18	23	24
Minimum	0.57	0.27	1.47	5.250	30.43	23.73	31.05	34.45	44.66
Maximum	2.69	4.3	8.78	17.91	56.48	51.55	59.59	51.49	53.77
Range	2.12	4.03	7.31	12.66	26.05	27.82	28.54	17.04	9.110
Mean	1.31	1.67	4.23	11.64	43.47	40.03	45.38	40.87	48.53
Std. Deviation	0.65	1.41	2.05	4.12	6.81	7.08	7.76	4.80	2.49
Std. Error of Mean	0.16	0.33	0.48	1.03	1.31	1.77	1.83	1.00	0.51
Relative Std. Deviation	49.94%	84.23%	48.37%	35.40%	15.67%	17.69%	17.09%	11.74%	5.12%
**Melamine laminate**									
RH (%)	25.14 ± 0.19	30.23 ± 0.14	40.03 ± 0.25	49.95 ± 0.23	55.17 ± 0.10	64.87 ± 0.34	75.20 ± 0.15	84.96 ± 0.52	N/A
Number of values	19	17	12	17	29	25	24	28	24
Minimum	0.37	0.7100	4.300	7.720	34.02	40.04	36.45	35.39	45.20
Maximum	0.76	2.840	9.270	28.97	68.88	79.95	72.47	70.13	53.26
Range	0.39	2.130	4.970	21.25	34.86	39.91	36.02	34.74	8.060
Mean	0.53	1.39	6.54	18.45	54.79	59.49	55.45	46.15	49.26
Std. Deviation	0.12	0.70	1.35	6.39	9.15	11.72	10.36	9.33	2.04
Std. Error of Mean	0.028	0.17	0.39	1.55	1.70	2.34	2.11	1.76	0.42
Relative Std. Deviation	22.98%	50.24%	20.64%	34.65%	16.70%	19.70%	18.67%	20.22%	4.14%

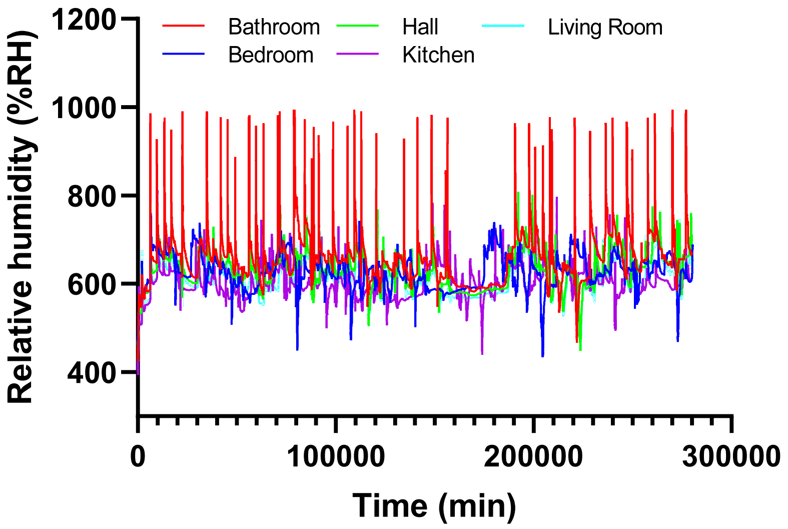

Figure S16. Humidity data for UK household (10th August – 19th September 2016). Taken from supplementary information of Pullinger et al., (2021). Y-axis scale requires division by 10 to give %RH.

9. Modelling the relationship between absolute humidity and transfer efficiency

The purpose of this supplementary section is to provide a detailed account of the development and quantification of a statistical model relating absolute humidity to transfer efficiency. This model was used to inform a quantitative microbial risk assessment analysis of the risk of exposure to SARS-CoV-2 and its dependence on environmental factors.

9.1 Parametrisation of transfer efficiency data

The data to which the model comprise 487 contact measurements at 15N with coupons of five materials: ABSS (n = 47), ABST (n = 53), Kydex (KYD, n = 59), melamine (MEL, n = 175) and stainless steel 304 (SS304, n = 153). For each AFP-coupon contact, the quantity of fluorescein on the finger (F) and coupon surfaces (C) were measured (and recorded in luminescent units), these measurements being adjusted to account for the background luminescence of the buffer solution. Alongside these are percentage RH, temperature (°C) and a record of the batch from which the AFP used in each measurement was taken.

The variations in temperature seen in the contact experiments, although within tight bounds, were seen to produce small but non-negligible variation in absolute humidity. Therefore, this latter quantity, rather than relative humidity, was thought to be a more appropriate predictor of transfer efficiency, and so forms the focus of the analysis here. Absolute humidity (g·m^{-3}) is related to temperature (°C) and percentage relative humidity via the following formula

$$ab.humidity = \frac{6.112 \times e^{\frac{17.67 \times temp}{temp+243.5}} \times rel.humidity \times 2.1674}{273.15 + temp}$$

E.g., a temperature of 19.9°C and relative humidity of 31% yields an absolute humidity of 5.33 g·m^3, whereas a temperature of 22.9°C at the same relative humidity yields 6.34 g·m^{-3}.

The response variable, Y, in the analysis is the ratio of fluorescein on the finger to the sum of the dye on both finger and that remaining on the coupon surface, post-contact, i.e.,

$$Y \stackrel{\text{def}}{=} \frac{F}{F + C}$$

This quantity Y is not a direct measurement of transfer efficiency, T, since the finger does not come into contact with the entire coupon area. Instead only the contaminant situated in a (variable) proportion of the coupon area, A, is available for transfer, so that

$$Y = T \times A$$

where we assume that fluorescein is spread evenly across the coupon surface. This assumption is appropriate considering consistency of aerosol deposition across coupons (Figure 2). Contact areas were measured (n=18) in separate experiments at a contact pressure of 15 N with mean and standard deviation of 233.08 and 27.94 mm^2. Figure S17 below is a quantile-quantile plot comparing the quantiles of these normalised measurements with the

corresponding quantiles of a standard normal distribution, suggesting that a normal prior with mean m_A = 0.1865 and standard deviation σ_A = 0.0224 is appropriate for the unobserved contact area proportions, A_i (since the coupons each have area 1250 mm^2. Note there is some variation in coupon area which is unaccounted in the models (Table S1).

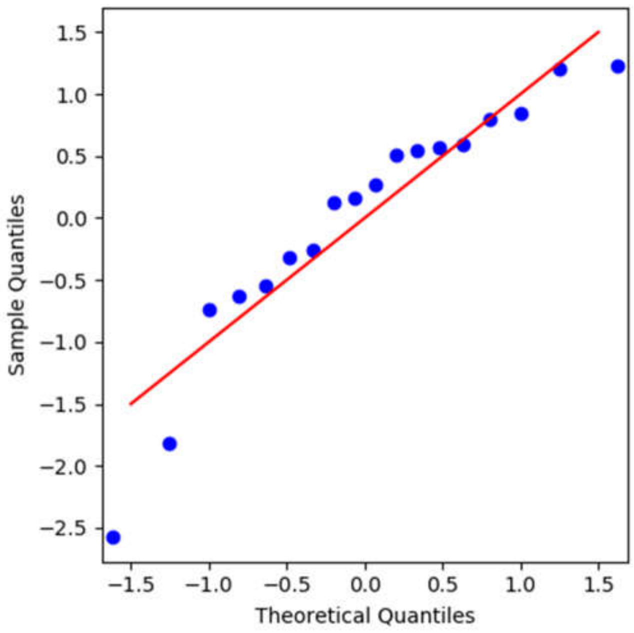

Figure S17. Quantile-quantile plot comparing quantiles of contact area measurements with those of standard normal distribution.

Therefore, the transfer efficiencies, T_i, corresponding to each individual contact are treated here as latent variables. Conditional on these, it is straightforward to show that each Y_i is normally distributed with mean $m_A T_i$ and standard deviation $\sigma_A T_i$, i.e.,

$$Y_i|T_i \sim Normal(m_A T_i, \sigma_A T_i)$$

where the index i runs over each transfer measurement.

9.2 Model descriptions

Each latent transfer efficiency, T_i, is modelled using a beta distribution (a natural and flexible choice for modelling proportions) with mean μ_i and precision ϕ_i, i.e.,

$$T_i|\mu_i, \phi_{m(i)} \sim Beta\left(\mu_i, \phi_{m(i)}\right)$$

where each material has its own precision parameter, ϕ, with m(i) denoting the coupon material (ABSS, ABST, KYD, MEL or SS304) corresponding to the ith observation. T_i therefore has probability density function

$$f_T(t|\mu, \phi) = \frac{1}{B(\mu\phi, (1-\mu)\phi)} t^{\mu\phi-1}(1-t)^{(1-\mu)\phi-1}$$

where

$$B(x,y) = \int_0^1 t^{x-1}\,(1-t)^{y-1}dt$$

is the beta function. In terms of the mean and precision, the variance of the beta distribution is:

$$\frac{\mu(1-\mu)}{\phi+1}$$

A number of candidate models are considered for the means, μ_i. The first model relates the μ_i to the absolute humidity via a sigmoidal transformation (logit link function) to a linear predictor with an intercept and slope term for each of the five materials. In addition, random effect for each of the 10 finger batches are included, which account for the apparent correlation in the Y_i among fingers taken from the same batch. These are assumed to be distributed independently as normal random variates with zero mean and standard deviation σ, which is to be estimated. Note this random batch effect is considered to be due to variation in applied sweat-sebum emulsion.

$$\mu_i = \frac{1}{1+e^{-\eta_i}} \qquad \text{(Model 1)}$$

$\eta_i = \beta_{0,m(i)} + \beta_{1,m(i)} \times AH_i + \tau_{b(i)}$, for i = 1, …., 487, and

$\tau_b \sim N(0,\sigma), b = 1,\dots,10$ (independently)

where, in the above, b(i) denotes the batch from which the artificial finger was taken and AH_i the absolute humidity for the ith observation. τ_b denotes the random effect corresponding to the bth finger batch.

$$\mu_i = \frac{M_{m(i)}}{1+e^{-\eta_i}} \qquad \text{(Model 2)}$$

Such a model implicitly assumes that for sufficiently high levels of absolute humidity, the transfer efficiency will approach unity. The second model includes a saturation level, $0 < M_{m(i)} < 1$, for each material representing the limiting level of transfer for large absolute humidity's. In symbols, this is:

A third model assumes that a common intercept, β_0, and slope, β_1, term are shared among the five materials, and a fourth model omits the intercept term altogether. Additionally, the four models are fitted without random batch effect. The models are summarised in Table S8.

9.3 Model fitting and choice of priors

Models were fitted using the Bayesian inference software Stan [67] that employs the No U-turn Sampler (NUTS) variant of Hamiltonian Monte Carlo [68] to sample from the posterior distributions of the parameters β_0, β_1, M, ϕ, and σ, latent transfer efficiencies, T, and random batch effects, τ, given the observations, Y. Diffuse independent marginal prior distributions were adopted for the parameters, expressing minimal knowledge about these quantities, as well as a lack of correlation between them, prior to observing the data. Each of the intercept and slope parameters (β_0, β_1) were assigned normal prior distributions with zero mean and a standard deviation of 100, the saturation level, M, was given a uniform distribution on its support, the unit interval, and finally the precision parameters, ϕ, and the random effect standard deviation, σ, were given exponentially distributed priors with rate 10^{-2}. To reduce posterior correlation between β_0, β_1, the absolute humidity's were mean-centred, i.e.,

$$AH_i^c = AH_i - \overline{AH}$$

where the means were taken over each of the five materials for Models 1 and 2 and over the entire data set for Models 3 and 4. For each of the eight models fitted, 20 000 samples in total were gathered using four independent sampler chains, with the first 2000 iterations (discarded before analysis) used for adaptation and burn-in. All of the sampler chains converged to the posterior distribution remarkably quickly, this being assessed by inspection of trace plots.

9.4 Model comparison and selection

The fitted models were compared using the idea of Bayesian p-values (BPV) [69]. These compare the posterior distribution of a chosen summary, S (Y, θ), of the data, Y, and model parameters, θ, drawn from the posterior distribution, with S (Y^{rep}, θ), where Y^{rep} are replicate data drawn from the posterior-predictive distribution

$$P(Y^{rep}|Y) = \int P\,(Y^{rep}|T)P(T|\theta)P(\tau|\theta)P(\theta|Y)d\theta$$

The choice of summary statistic, S, is model and context-dependent and is typically selected to target particular aspects of a model wished to be tested, by way of comparing a model's predictions with the data to which it is fitted. In our case, we wish to inspect squared distances of the observations, Y, from the fitted means, μ, and accounting for the fact that the variance of the (unobserved) transfer efficiencies is not constant. Therefore:

$$S(Y,\mu) = \sum \frac{(Y_i - \mu_i)^2(\phi_{m(i)} + 1)}{\mu_i(1 - \mu_i)}$$

which is the sum of squared distances of each observation Y_i from model's estimate of the mean transfer, μ_i, normalised by the estimated variance (see above). The Bayesian p-value is:

$$P(S(Y^{rep},\theta) > S(Y,\theta)|Y)$$

which is the posterior probability that the predicted values of S are at least as large as those derived from the data. For a model that fits reasonably well, it is expected that the BPV is

close to one half, with values close to zero or one indicating that the model could be improved upon.

The BPVs are given in Table S8, where the values marked with an asterisk correspond to the model fitted without the random finger batch effect. By a clear margin Model 1 performs the poorest, suggesting that the model fit is greatly improved by addition of a saturation term for each of the five materials. Models 2 and 3 show comparable performance, with both appearing to fit well to the data. This is in spite of Model 3's reduced number of parameters, since the intercept and slope terms are shared across all materials. A limitation of BPVs is that they do not penalise models with more parameters, in contrast to the various information criterion measures of model fit, such as AIC, BIC etc. Even though Model 4's BPV compares well with Model 2 and 3's, inspection of plots (see https://github.com/leeds-indoor-air/touch_tx) show that the intercept terms are unlikely to be zero, and so this model is rejected. Therefore, Model 3, with batch effect, is our preferred model. Table S9 contains summary posterior statistics for each of the parameters of the fitted model.

9.5 Marginal posterior densities for the fitted model

Figure S18 below summarises the marginal posterior densities for each parameter of the fitted model. Additional plots, summarising correlations between certain pairs of parameters, as well as posterior-predictive plots (similar to Figure S18) for the other fitted models can be found at the link https://github.com/leeds-indoor-air/touch_tx.

Equation (2) in the main manuscript implies a sigmoid relationship between AH and mean transfer efficiency. This means that, for low AH, transfer is expected to be close to zero and, as AH increases, so does the expected transfer, which eventually saturates at $M_{material}$. The rate of increase of mean transfer is at its greatest when $\eta = 0$, i.e., when

$$AH_0 = \frac{-(\beta_0 + \tau_b)}{\beta_1}$$

The parameters β_0 and β_1 have posterior medians -7.401 (95% credible interval (CI): [-7.960, -6.868]) and 0.738 (95% CI: [0.681, 0.798]), and therefore the model predicts that, for a batch effect of zero, AH_0 is approximately 10 gm^{-3} for each of the coupon materials. The five coupon materials do differ, however, in terms of the expected level of transfer at large AH. The density plots in Figure 1 clearly suggest that the material-specific parameters, $M_{material}$, for the five materials are all different with ABSS exhibiting the highest transfer efficiency for large AH, and ABST the lowest. There is evidence that the precision parameters, $\phi_{material}$, are also material-dependent. The greater degree of posterior uncertainty in the estimates of ϕ_{ABSS}, ϕ_{ABST} and ϕ_{KYD}, (demonstrated by their large variances) could possibly be due, however, to there being fewer replicate experiments performed for these three materials than for melamine and stainless steel 304. Finally, the random effect standard deviation, σ, has posterior median 0.223 (95% CI: [0.070, 0.500]), meaning that for fixed β_0 and β_1:

$$AH_0 \sim Normal\left(-\frac{\beta_0}{\beta_1}, 0.223\right)$$

and so a non-negligible amount of the overall variability in transfer efficiency recorded in the experiments is due to variations in transfer between the finger batches. This supports experimental

observations where although finger topology is well controlled from batch to batch, the application of sweat-sebum emulsion is not and a recommendation is to improve that process to reduce variation.

Table S8. Summary of fitted models and Bayesian p-value statistics.

Model†	Model term		
	μ_i	η_i	BPV
1	$\mu_i = \frac{1}{1+e^{-\eta_i}}$	$\eta_i = \beta_{0,m(i)} + \beta_{1,m(i)} AH_i + \tau_{b(i)}$ $\tau_b iid \sim Normal(0, \sigma), b = 1, \ldots, 10$	0.128, 0.043*
2	$\mu_i = \frac{M_{m(i)}}{1+e^{-\eta_i}}$	$\eta_i = \beta_{0,m(i)} + \beta_{1,m(i)} AH_i + \tau_{b(i)}$	0.454, 0.458*
3		$\eta_i = \beta_0 + \beta_1 AH_i + \tau_{b(i)}$	0.448, 0.430*
4		$\eta_i = \beta_1 AH_i + \tau_{b(i)}$	0.412, 0.304*

†= Each of the four models were fitted with and without the random batch effects, τ_b. Bayesian p-values for the model versions without random batch effect are indicated with an asterisk.

Table S9. Posterior parameter summary statistics (to 3 decimal places) for Model 3

Parameter	Statistical factors for parameter				
	Mean	Standard deviation	2.5%-tile	Median	97.5%-ile
β_0	-7.405	0.279	-7.960	-7.401	-6.868
β_1	0.739	0.030	0.681	0.738	0.798
σ	0.239	0.110	0.070	0.223	0.500
**ABSS**					
M_{ABSS}	0.652	0.025	0.606	0.652	0.703
ϕ_{ABSS}	78.470	31.398	35.630	72.426	155.860
**ABST**					
M_{ABST}	0.307	0.014	0.282	0.307	0.335
ϕ_{ABST}	60.274	14.338	36.667	58.765	92.504
**Kydex**					
M_{KYD}	0.376	0.014	0.350	0.376	0.403
ϕ_{KYD}	82.995	18.895	51.756	80.979	125.710
**Melamine**					
M_{MEL}	0.574	0.016	0.542	0.574	0.607
ϕ_{MEL}	13.922	1.761	10.776	13.817	17.647
**Stainless steel 304**					
M_{SS304}	0.450	0.014	0.424	0.450	0.477
ϕ_{SS304}	28.256	3.920	21.358	27.981	36.703

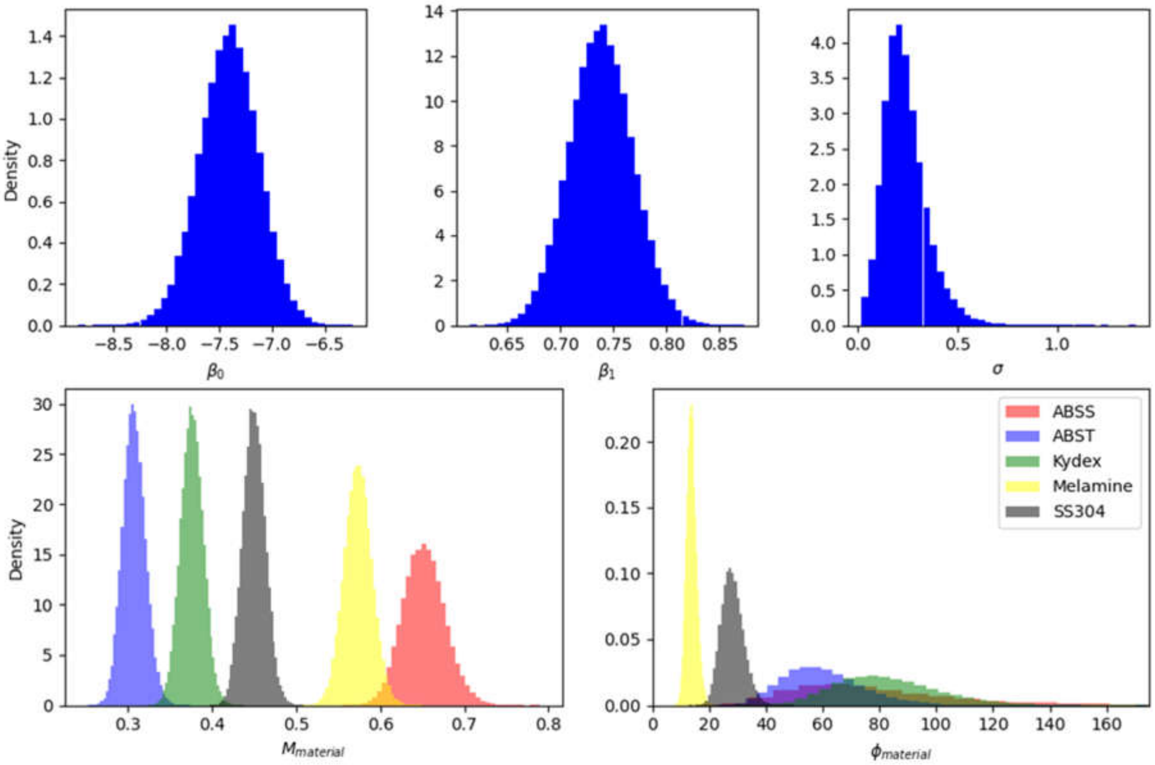

Figure S18. Marginal posterior density histograms of all fitted model parameters.

The contents include material subject to © Crown copyright (2022), Dstl. This material is licensed under the terms of the Open Government Licence except where otherwise stated. To view this licence, visit http://www.nationalarchives.gov.uk/doc/open-government-licence/version/3 or write to the Information Policy Team, The National Archives, Kew, London TW9 4DU, or email: psi@nationalarchives.gsi.gov.uk
